# Mental health impact on the unmet need for family planning and fertility rate in rural Ethiopia: a population-based cohort study

**DOI:** 10.1017/S2045796020000736

**Published:** 2020-08-18

**Authors:** R. Catalao, G. Medhin, A. Alem, M. Dewey, M. Prince, C. Hanlon

**Affiliations:** 1 Academic Clinical Fellow in Psychiatry, South London and Maudsley NHS Foundation Trust and King's College London, London, UK; 2 Aklilu-Lemma Institute of Pathobiology, Addis Ababa University, Addis Ababa, Ethiopia; 3 Department of Psychiatry, WHO Collaborating Centre for Mental Health Research and Capacity-Building, School of Medicine, College of Health Sciences, Addis Ababa University, Addis Ababa, Ethiopia; 4 Centre for Global Mental Health, Health Service and Population Research Department, Institute of Psychiatry, Psychology and Neuroscience, King's College London, London, UK; 5 King's Global Health Institute, King's College London, London, UK; 6 Centre for Innovative Drug Development and Therapeutic Trials for Africa (CDT-Africa), College of Health Sciences, Addis Ababa University, Addis Ababa, Ethiopia

**Keywords:** Contraception, family planning, fertility, mental health

## Abstract

**Aims:**

Although much research has focused on socio-demographic determinants of uptake of contraception, few have studied the impact of poor mental health on women's reproductive behaviours. The aim of this study was to examine the impact of poor mental health on women's unmet need for contraception and fertility rate in a low-income country setting.

**Methods:**

A population-based cohort of 1026 women recruited in their third trimester of pregnancy in the Butajira district in rural Ethiopia was assessed for symptoms of antenatal common mental disorders (CMDs; depression and anxiety) using Self-Reporting Questionnaire-20. Women were followed up regularly until 6.5 years postnatal (between 2005 and 2012). We calculated unmet need for contraception at 1 year (*n* = 999), 2.5 (*n* = 971) and 3.5 years (*n* = 951) post-delivery of index child and number of pregnancies during study period. We tested the association between CMD symptoms, unmet need for contraception and fertility rate.

**Results:**

Less than one-third of women reported current use of contraception at each time point. Unmet need for birth spacing was higher at 1 year postnatal, with over half of women (53.8%) not using contraception wanting to wait 2 or more years before becoming pregnant. Higher CMD symptoms 1 year post-index pregnancy were associated with unmet need for contraception at 2.5 years postnatal in the unadjusted [odds ratio (OR) 1.09; 95% confidence interval (CI) 1.04–1.15] and fully adjusted model [OR 1.06; 95% CI 1.01–1.12]. During the 6.5 year cohort follow-up period, the mean number of pregnancies per woman was 2.4 (s.d. 0.98). There was no prospective association between maternal CMD and number of pregnancies in the follow-up period.

**Conclusions:**

CMD symptoms are associated with increased unmet need for family planning in this cohort of women with high fertility and low contraceptive use in rural Ethiopia. There is a lack of models of care promoting integration of mental and physical health in the family planning setting and further research is necessary to study the burden of preconception mental health conditions and how these can be best addressed.

## Introduction

Family planning has been shown to improve women's and children's lives in a multitude of ways (Ahmed *et al*., 2012; Cleland *et al*., 2012). Although much research to date has focused on socio-economic determinants of the uptake of family planning (Tadele *et al*., 2019), few investigators have studied the impact of poor mental health on women's reproductive behaviours in low- and middle-income countries (LMICs). There is evidence from high-income countries that depression is associated with increased rates of unintended pregnancy in young adulthood and shorter pregnancy spacing (Patchen *et al*., 2009; Hall *et al*., 2014; Corcoran, 2016). Depression and stress have also been found to be associated with lower rates of contraceptive use and choice of less effective methods, mostly in clinical samples from North America (Garbers *et al*., 2010; Hall *et al*., 2013a, b). Despite the high prevalence of common mental disorders (CMDs) in women of reproductive age in LMICs (Fisher *et al*., 2012) and mounting evidence of the impact of CMDs on infant illness rates (Rahman *et al*., 2004; Ross *et al*., 2011), and reduced help-seeking of the mother on behalf of her child (Rahman *et al*., 2004), less focus has been given to how maternal CMDs may impact a woman's behaviours pertaining to her own health.

Ethiopia embraced the Family Planning 2020 commitment (Family Planning, 2020), aiming to increase contraceptive use prevalence to 55% and reach an additional 6.2 million women by 2020 (Brown *et al*., 2014). Although evidence shows that the rates of family planning uptake were increasing rapidly in Ethiopia pre-FP2020 pledge, the growth rate seems to have stalled in recent years (Ahmed *et al*., 2019). In the Ethiopian Demographic Health Survey (DHS) 2016, 22% of married women reported unmet need for family planning (CSA Addis Ababa and ICF, 2016); the number was higher in rural than in urban areas and there were wide regional and socio-demographic variations. The follow-up mini DHS 2019 showed a contraceptive prevalence rate of 41%, an improvement from 35% in 2016 but still below the FP2020 pledge (EPHI and ICF, 2019). Previous population studies in Ethiopia have shown that CMD symptoms are common in the perinatal period and are associated with impaired maternal functioning (Senturk *et al*., 2012), however to our knowledge, no previous studies have explored if CMD symptoms impact women's uptake of family planning.

We therefore aimed to investigate (1) if CMD symptoms were prospectively associated with unmet need for contraception and (2) if high CMD symptoms impacted fertility rate in a cohort of women in rural Ethiopia.

## Methods

**Fig. 1.**
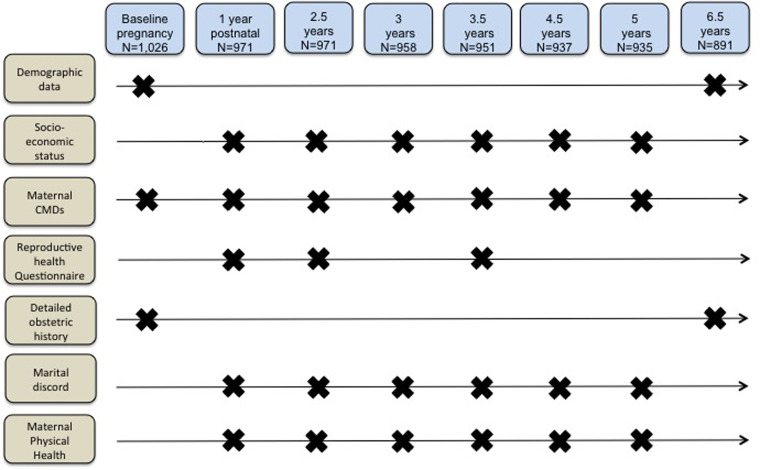
Assessment time-points.

This cohort study is part of the C-MaMiE project (*C*hild health, growth and development in relation to *ma*ternal *me*ntal disorder *i*n *E*thiopia), a population-based sample of 1065 pregnant women recruited between July 2005 and March 2006 (Hanlon *et al*., 2009a, b) and followed up at 12 months postpartum, 2.5 years and at regular intervals of 6–12 months up to 6.5 years (Fig. 1). The primary aim of the project was to evaluate the public health impact of maternal CMD upon infant and child outcomes; this study was a hypothesis-driven secondary analysis.

### Study setting

The study was situated in the health and demographic surveillance site (HDSS) at Butajira, a predominantly rural area of Ethiopia located 130 km south of the capital city, Addis Ababa, in the Gurage Zone, Southern Nations, Nationalities and Peoples’ Region (SNNPR) of Ethiopia (Berhane *et al*., 1999). Fertility levels in this region remain high, with a total fertility rate of 4.4 births per woman in 2016 (CSA Addis Ababa and ICF, 2016), compared to 5.6 in 2005, at the time of the start of our study (CSA Addis Ababa and ORC Macro, 2006). Modern contraceptive use in SNNPR was estimated to be 11.9% in 2005, which has increased to 44.6% among married women age 14–49 years in 2019 (EPHI and ICF, 2019). Over the past decade, the Ethiopian government has invested in the Health Extension Programme (HEP) with a strong emphasis on improving rural health care services by task sharing and capacity building (Ethiopia FMOH, 2012a). Two female health extension workers (HEWs), who receive 1 year of training on 16 essential health packages including maternal and child health, family planning and adolescent reproductive health, staff health posts serving a population ranging from 3000 to 5000 in a village (*kebele*) (Ethiopia FMOH, 2005). The programme has a strong community focus. HEWs spend a substantial amount of time on home visits and train model families on HEP health packages so these households are able to implement their knowledge and influence neighbours to adopt these practices (Wang *et al*., 2016).

### Study participants

For the original cohort, eligible women were between the ages of 15 and 49 years, able to converse in Amharic (the national language of Ethiopia), living in the HDSS and in the third trimester of pregnancy during the study recruitment period (Hanlon *et al*., 2009a, b). For the current study, we restricted our data analysis to women aged between 15 and 38 at recruitment so that all would be within the reproductive window throughout the study period [*n* = 1026 (96.3% of original cohort)]. The women were identified by the Butajira Rural Health programme (BRHP) enumerators in the course of their 3-monthly surveillance interviews and, after giving informed consent, were interviewed by the project data collectors (Berhane *et al*., 1999).

### Measures

#### Maternal CMDs

Maternal CMD was measured at baseline, 12 months postpartum and at five other time points up to 6.5 years post-recruitment (Fig. 1) using Self-Reporting Questionnaire, 20-item version (SRQ-20). The SRQ is a 20-item scale that asks about depressive, anxiety and somatic symptoms present in the preceding month, generating a continuously distributed scale score indicating the level of overall psychological morbidity. The SRQ-20 was extensively validated for use in a mixed sample of pregnant and postnatal women in the Butajira population (Hanlon *et al*., 2008).

#### Reproductive health behaviours and pregnancy

The project data collectors administered a reproductive health questionnaire at 12 months, 2.5 years and 3.5 years post baseline (Fig. 1). The questionnaire covered questions on current pregnancy status, pregnancy spacing intentions, knowledge of specific contraceptive methods, current use and reasons for not using contraception. Detailed parity information was collected at baseline and at 6.5 years. Number of pregnancies per woman during this cohort study was calculated by subtracting initial parity from total number of pregnancies reported at 6.5 years.

#### Unmet need for family planning

We adapted the definitions of unmet need for contraception from the Guide to DHS Statistics (Croft *et al*., 2018). Unmet need for limiting was defined as the percentage of women who reported they did not want to have more children and were neither pregnant nor using contraception at the time. Unmet need for spacing was operationalised as the percentage of women not using contraception and not pregnant who answered they would like to wait 2 years or more for the next pregnancy. At each time point, some women were pregnant but reported they did not want to be pregnant. Their numbers have been presented as a separate column in the unmet need for contraception. Women pregnant due to contraceptive failure were not included. Total unmet need for family planning was, therefore, defined as the proportion of women who were not pregnant and wanted to postpone their next birth for 2 or more years or stop childbearing altogether, but were not using a contraceptive method or had a mistimed or unwanted current pregnancy.

The denominator for unmet need was the total number of women completing the reproductive health questionnaire in each cohort time point. A further condition for unmet need is that women are fertile. All women in our cohort were in their third trimester of pregnancy at recruitment and their upper age limit was restricted to 38 years.

#### Confounders

Confounders were selected on the basis of published evidence (CSA Addis Ababa and ICF, 2016). Socio-demographic variables including age, marital status, religion, maternal and paternal educational level were collected at baseline.

A composite variable measuring socio-economic status (SES) was created by adding the following poverty indicators: self-reports of indebtedness, hunger in the last month and perceived lower relative wealth (Hanlon *et al*., 2009a, b). This was measured at baseline and at five other time points. The number of times the woman reported two or more poverty indicators was calculated across the sample.

Similarly, marital discord was indicated through self-report that (i) the husband was not providing enough help, (ii) the relationship was ‘average, bad or very bad’, (iii) they sometimes or often quarrelled or (iv) the woman felt that her husband drank too much alcohol (Servili *et al*., 2010). Poor physical health was defined as a self-reported episode of malaria, diarrhoea or fever measured for the 6 months preceding each time point (Smartt *et al*., 2016). The number of time points with the described measures were added across the whole sample (Fig. 1).

### Analyses

Stata version 15 software was used for data analysis (StataCorp, 2017). The initial analysis strategy was hypothesis-driven, using logistic regression to investigate the association between (1) CMD symptoms (SRQ20 as continuous variable) at 12 months upon total unmet need for family planning as defined above at 2.5 years and between (2) CMD symptoms (SRQ 20 as continuous variable) at 3 years and unmet need for contraception at 3.5 years. We separated the analyses for these time points because the time interval between exposure and outcome was different and there could be different patterns of associations over the time since the index pregnancy. These hypotheses were partially and fully adjusted to confounders identified *a priori*, including demographic and socio-economic characteristics. A further exploratory model calculated crude and adjusted odds ratio (aOR) for the effect of these other variables on unmet need for contraception.

In addition, simple and multiple Poisson regression was used to test the hypothesis that maternal CMD would be associated with higher number of cumulative pregnancies during the project follow-up period. A fully adjusted exploratory model calculated incidence rate ratios (IRRs) with the corresponding confidence intervals (CIs) for the effects of other variables, including contraceptive use, socio-demographic and obstetric factors on the number of pregnancies the during study period. Missing data were addressed by case-wise deletion.

## Results

Follow-up rates were 94.6% (*n* = 971) at 2.5 years, 92.7% (*n* = 951) at 3.5 years and 86.8% (*n* = 891) at 6.5 years (Fig. 1). Women who were lost to follow-up did not differ by age, religion or husband's education, but were less likely to have formal education and more likely to have a lower socio-economic score and higher CMD symptoms at baseline (Table 1).

**Table 1.** Characteristics of the cohort

Characteristic		Number at baseline (%) *n* = 1026	Number at 6.5 years (%) *n* = 891
Age (years)		Mean 26.3 (s.d. ±5.9)	Mean 33.4 (s.d. ±5.9)
Maternal formal education		215 (21.0%)	146 (16.4%)*
Husband education (primary and above)		652 (64.0%)	523 (60.6%)
Composite SES score (2 or more poverty indicators)		159 (15.5%)	65 (7.5%)**
Religion			
Orthodox Christian		156 (15.2%)	133 (15.0%)
Muslim		794 (77.4%)	690 (77.4%)
Catholic/Protestant		76 (7.4%)	68 (7.6%)
Marital status			
Monogamous marriage		82.9 (80.8%)	713 (80.0%)
Polygamous marriage		187 (18.2%)	150 (16.8%)
Parity		Mean 3.0 (s.d. 2.3) Min 0, max 11	Mean 5.5 (s.d. 2.1)** Min 1, max 13
High CMD symptoms		119 (1.6%)	46 (5.2%)**
SRQ total score	At 12 months (*n* = 999)	Median 3 (25th centile 0, 75th centile 3) SRQ ≥ 6, *N* = 99	
	At 3 years (*n* = 958)	Median 1 (25th centile 0, 75th centile 2) SRQ ≥ 6, *N* = 41	
Characteristics across cohort study period (*n* = 891)			
		1 time point	2 time points
High CMD symptoms (SRQ ≥ 6)		134 (15.0%)	58 (6.5%)
Low SES score		164 (18.4%)	139 (15.6%)
Marital discord		251 (28.2%)	357 (40.1%)
Poor physical health		374 (42.0%)	381 (42.8%)
Number of pregnancies per woman (*n* = 876)		Mean 2.4 (s.d. 0.98) range 0–6	

Significant difference between characteristic at baseline and 6.5 years.

*p < 0.05; **p < 0.01.

Mean maternal age for women at time of recruitment into the study was 26.3 years (s.d. ±5.9) at baseline and almost all women were married (99.0%), with 18.2% at baseline and 16.8% at 6.5 years in a polygamous marriage. Only 21.0% of women at the time of the index pregnancy had formal education, whereas over 50% of husbands had attended primary school or above. Over two-thirds of women were Muslim. At baseline mean parity was 3.0 (s.d. ±2.3) whereas at 6.5 years parity was 5.5 (s.d. ±2.1).

Two or more poverty indicators were endorsed by 15.6% women at two or more time points. Marital discord was reported by 40.1% of women at two or more time points and 42.8% reported recent poor health at two or more time points (Table 1).

High levels of CMD symptoms (SRQ ≥ 6) were experienced by 15.0% of women at one time point and 6.5% at two or more time points during the follow-up period. At 1 year postnatal, 43 women (4.3% of total sample) were pregnant again. At 2.5 years and 3.5 years, 24.3 and 14.8% of women reported current pregnancy, respectively. Of these, 37.2% at 12 months, 48.7% at 2.5 years and 27.7% at 3.5 years were unwanted pregnancies and 20.8% at 2.5 years and 17.7% at 3.5 years were due to contraceptive failure (Table 2). Some women became pregnant while using a long-acting modern contraceptive such as injection: 16.5% of all pregnancies at 2.5 years (*n* = 39); 14.2% of all pregnancies at 3.5 years (*n* = 20).

**Table 2.** Reproductive health questions (number of affirmative answers/total sample)

	12 months (*n* = 999)	2.5 years (*n* = 971)	3.5 years (*n* = 951)
Currently pregnant	43 (4.3%)	236 (24.3%)	141 (14.8%)
Unwanted pregnancy	16/43 (37.2%)	115/236 (48.7%)	39/141 (27.7%)
Using contraception when conceived	–	49/236 (20.8%)	25/141 (17.7%)
Using OCP tablets	–	10/236 (4.2%)	5/141 (3.5%)
Using injection	–	39/236 (16.5%)	20/141 (14.2%)
Not pregnant and using contraception	291 (29.1%)	243 (25.0%)	201 (21.1%)
Unmet need for contraception [not using contraception and (a); (b); (c)]			
(a) Want to wait 2+ years to become pregnant (for spacing)	538 (53.8%)	326 (33.6%)	351 (36.9%)
(b) Does not want to have more children (for limiting)	88 (8.8%)	78 (8.0%)	144 (15.4%)
(c) Current mistimed/unwanted pregnancy	13 (1.3%)	85 (8.8%)	26 (2.7%)
Total (a + b + c)	639 (64.0%)	489 (50.4%)	521 (54.8%)
Awareness of contraceptive methods			
OCP	948 (94.9%)	968 (99.7%)	949 (99.8%)
Injection	965 (96.6%)	969 (99.8%)	948 (99.7%)
Coil	117 (11.7%)	177 (18.2%)	193 (20.3%)
Condom	332 (33.2%)	432 (44.5%)	451 (47.4%)
Sterilisation	63 (6.3%)	110 (11.3%)	129 (13.6%)
Reason for not using contraception			
Prefer to leave it to nature	330 (33.0%)	248 (25.5%)	262 (27.5%)
Want another child	148 (14.8%)	62 (6.4%)	50 (5.3%)
Health reasons	41 (4.1%)	71 (7.3%)	80 (8.4%)
Husband wants another child/does not allow me	16 (16.0%)	7 (7.2%)	11 (11.6%)
Religion does not allow	8 (8.0%)	–	2 (2.1%)
No available family planning	5 (5.0%)	2 (2.0%)	–
Husband approves contraception	691 (69.2%)	644 (66.3%)	652 (68.6%)

### Unmet need for contraception

Less than one-third of women reported current use of contraception at each of the time points. Unmet need for spacing was higher at 12 months, with over half of women (*n* = 538, 53.8%) not using contraception and wanting to wait for 2 or more years before the next pregnancy. Unmet need for contraception for limiting was higher at 3.5 years when 206 women (21.6%) reported not wanting to have more children. Total rates of unmet need for contraception were calculated to be 64.0% at 12 months, 53.1% at 2.5 years and 62.7% at 3.5 years (Table 2).

Most women were familiar with modern contraceptive methods and over 95% of women knew about the oral combined pill and long-acting injectables. Less than half reported familiarity with condoms. The most commonly reported reasons for not using contraception was a preference to leave it to nature (33.0% at 12 months, 25.5% at 2.5 years and 27.5% at 3.5 years). Only a very small proportion reported not using contraception for religious reasons or lack of access (<5%). More than two-thirds of women reported that their husbands approved of contraception.

### Effect of CMD symptoms on unmet need for contraception

CMD symptoms were higher at 12 months (median SRQ score 3, 25th centile 0, 75th centile 3, *n* = 99) than at 3 years (median SRQ score 1, 25th centile 0, 75th centile 2, *n* = 41) and other subsequent points in the study period. Higher levels of CMD symptoms at 12 months post-index pregnancy were associated with higher levels of unmet need for contraception at 2.5 years in both the crude (OR 1.09; 95% CI 1.04–1.15 for every 1 point increase in SRQ score) and fully adjusted hypothesis-driven models (aOR 1.06; 95% CI 1.01–1.12), including potential confounders of maternal and husband education, SES, age, parity at baseline and religion. However, higher levels of CMD symptoms at 3 years were not associated with unmet need for contraception at 3.5 years in either the unadjusted or adjusted model (Table 3).

**Table 3.** Hypothesis-driven model of the effect of CMD symptoms on unmet need for family planning at 2.5 and 3.5 years

	Unmet need at 2.5 years: OR (95% CI)	Unmet need at 3.5 years: OR (95% CI)
Unadjusted effect of CMD symptoms^a^	1.09 (1.04–1.15)***	0.96 (0.90–1.01)
Effect of CMD symptoms partially adjusted for:		
Maternal education	1.09 (1.03–1.14)***	0.96 (0.90–1.01)
Husband education	1.09 (1.04–1.14)***	0.96 (0.90–1.01)
Parity at baseline	1.08 (1.03–1.14)**	0.95 (0.90–1.01)
Polygamous marriage	1.09 (1.03–1.14)***	0.95 (0.90–1.01)
Religion	1.09 (1.04–1.15)***	0.96 (0.91–1.01)
SES	1.06 (1.01–1.12)*	0.94 (0.89–1.00)
Age	1.08 (1.03–1.14)**	0.95 (0.90–1.01)
Marital discord	1.08 (1.03–1.14)***	0.95 (0.90–1.01)
Poor physical health	1.08 (1.03–1.14)**	0.96 (0.90–1.02)
Fully adjusted effect of CMD symptoms^a^	1.06 (1.01–1.12)*	0.94 (0.87–1.01)

a CMD symptoms at 12 months for 2.5 year model and at 3 years for the 3.5 year model.

*p ≤ 0.05; **p ≤ 0.01; ***p ≤ 0.001.

In the fully adjusted exploratory model, only CMD symptoms and lower SES (OR 1.79; 95% CI 1.19–2.70) continued to be significant (Table 4).

**Table 4.** Multivariable model for factors associated with cumulative number of pregnancies during study period

	Unadjusted IRR and 95% CI	Adjusted^a^ IRR and 95% CI
High CMD symptoms at 1 time point	0.92 (0.81–1.04)	0.97 (0.85–1.11)
High CMD symptoms at 2 or more time points	0.99 (0.83–1.18)	1.06 (0.88–1.27)
Maternal education	0.93 (0.83–1.04)	0.94 (0.83–1.06)
Paternal education	1.02 (0.94–1.12)	1.03 (0.94–1.13)
Religion (Orthodox Christian as reference)		
Muslim	1.14 (1.01–1.30)*	1.14 (1.00–1.30)*
Catholic/Protestant	1.21 (0.99–1.47)*	1.22 (1.01–1.49)*
Parity at baseline (0–1 as reference)		
2–4	0.88 (0.80–0.98)*	0.95 (0.83–1.08)
≥5	0.72 (0.64–0.81)***	0.83 (0.68–1.01)
Polygamous marriage	0.88 (0.78–0.99)*	0.92 (0.81–1.04)
Marital discord at 1 time point	0.99 (0.88–1.10)	0.99 (0.88–1.10)
2+ time points	0.97 (0.87–1.07)	1.04 (0.93–1.16)
Reporting contraception use at 1–2 time points	0.96 (0.88–1.06)	0.91 (0.83–1.00)
Reporting contraception use at 3 time points	0.88 (0.73–1.06)	0.82 (0.68–0.99)*
Low SES score		
1 time point	0.96 (0.86–1.08)	0.95 (0.84–1.07)
2+ time points	0.99 (0.88–1.12)	1.00 (0.88–1.14)
Age	0.98 (0.97–0.99)***	0.99 (0.97–1.00)*
Poor self-reported physical health		
1–2 time points	0.98 (0.87–1.12)	1.00 (0.88–1.14)
3 or more time points	0.94 (0.83–1.06)	0.98 (0.86–1.12)

*p ≤ 0.05; **p ≤ 0.01; ***p ≤ 0.001.

a Adjusted model = multiple Poisson regression with all variables included.

### Cumulative number of pregnancies

During the 6.5 year follow-up period the mean number of pregnancies per woman (*n* = 876) was 2.4 (s.d. 0.98), ranging from 0 to 6 pregnancies (Table 2). There was no association between maternal CMD and number of pregnancies over the follow-up period in either the univariate or multivariable models [adjusted IRR (adj IRR) 0.97; 95% CI 0.85–1.11 for high CMD at one time point, aOR 1.06; 95% CI 0.88–1.27 at two or more time points]. In the fully adjusted multivariable model, consistent use of contraception led to a decrease of 18% in the number of pregnancies (adj IRR 0.82; 95% CI 0.68–0.99), and the effect of age was a decrease of 1% per year (adj IRR 0.99; 95% CI 0.97–1.00) (Table 5).

**Table 5.** Exploratory model for factors affecting unmet need for family planning at 2.5 and 3.5 years

	Unmet need at 2.5 years: ratio 95% CI		Unmet need at 3.5 years: ratio 95% CI	
Unadjusted effect of		Fully adjusted effect^b^	Unadjusted effect	Fully adjusted effect^b^
CMD symptoms^a^:	1.09 (1.04–1.15)***	1.06 (1.01–1.12)*	0.96 0.90–1.01)	0.94 (0.87–1.01)
Maternal education	0.92 (0.67–1.26)	1.12 (0.78–1.61)	0.69 (0.51–0.95)*	0.73 (0.51–1.04)
Husband education	0.77 (0.59–1.01)	0.82 (0.61–1.09)	0.87 (0.67–1.14)	1.00 (0.75–1.35)
Parity at baseline				
2–4	1.34 (0.99–1.81)	1.22 (0.81–1.85)	1.28 (0.94–1.73)	1.35 (0.89–2.04)
5+	1.42 (1.02–1.98)*	1.22 (0.66–2.24)	1.27 (0.93–1.81)	1.52 (0.82–2.81)
Polygamous marriage	1.42 (1.02–1.98) *	1.18 (0.81–1.71)	1.21 (0.86–1.70)	1.01 (0.70–1.47)
Religion (Orthodox Christian as ref)				
Muslim	0.95 (0.66–1.35)	1.08 (0.72–1.60)	1.62 (1.13–2.33)**	1.50 (1.01–2.23)*
Catholic or Protestant	0.62 (0.35–1.09)	0.67 (0.36–1.24)	0.82 (0.46–1.45)	0.71 (0.39–1.32)
Low SES				
1 time point	1.71 (1.20–2.43)**	1.61 (1.21–2.32)**	1.05 (0.99–1.03)	1.04 (0.73–1.49)
2+ time points	2.07 (1.41–3.05)***	1.79 (1.19–2.70)**	1.26 (0.86–1.83)	1.44 (0.95–2.18)
Age	1.03 (1.01–1.05)*	1.10 (0.97–1.05)	1.01 (0.69–1.52)	0.99 (0.96–1.04)
Marital discord				
1 time point	1.27 (0.90–1.78)	1.22 (0.85–1.73)	1.17 (0.83–1.65)	1.19 (0.83–1.70)
2+ time points	1.25 (0.91–1.70)	0.99 (0.71–1.40)	1.06 (0.77–1.45)	1.05 (0.75–1.48)
Poor physical health	1.20 (0.92–1.55)	1.02 (0.76–1.37)	1.02 (0.69–1.52)	0.96 (0.64–1.45)

a CMD symptoms at 12 months for 2.5 year model and at 3 years for the 3.5 year model.

b Fully adjusted model includes all factors in the table.

*p ≤ 0.05; **p ≤ 0.01; ***p ≤ 0.001.

## Discussion

### Main findings

In our study we found that high CMD symptoms at 1 year postnatal were associated with higher unmet contraceptive need at 2.5 years. This result was significant even after adjusting for other confounders known to traditionally impact adoption of modern contraception including maternal education, SES, parity and age (Tadele *et al*., 2019). An association was not seen between high CMD symptoms at 3 years and unmet contraceptive need at 3.5 years. There was also no association between CMD symptoms and number of pregnancies during the follow-up period.

We also found that unmet need for contraception was high in rural Ethiopia (range 56.3–64.0% in our cohort), particularly in the first year post-delivery (Borda and Winfrey, 2010 ; Tegegn *et al*., 2017). Our findings also highlight the dynamic nature of women's reproductive needs over time. Although at 12 months post recruitment the larger amount of need was for spacing (53.8%), the number of mistimed and unwanted pregnancies peaked at 2.5 years (11.5%) and the need for limiting increased at 3.5 years (15.4%) compared to the two previous time points. Importantly, around one-fifth of women became pregnant despite adoption of modern contraception (20.8% at 2.5 years and 17.7% at 3.5 years).

### Strengths and limitations

Strengths of our study include the large, representative sample of women from a rural setting in a low-income country, the use of locally validated measures of CMD and the prospective design with high rates of follow-up. Although previous studies have focused on child outcomes, the impact of CMDs on women's health behaviours has been largely neglected in published literature. To the best of our knowledge, this study is the first from a low-income country to examine impact of mental health on reproductive behaviours, with potential generalisability to other similar settings. Our study also has several potential limitations. There was differential attrition of women with higher baseline CMD and poverty. We expect that the association between maternal CMD and family planning might be higher in this group, although it is not possible to know the direction of any selection bias. Data collection for our cohort spanned from 2005 to 2012, which preceded the latest DHS survey of 2019, and therefore would not have captured the increase in contraceptive use that has occurred in the last few years. The observed association may, therefore, have changed as mental health interacts with different emerging barriers to uptake of family planning. However, our expectation is that women's mental health will become more salient as more structural barriers to access diminish in importance.

Our study is limited by the reliance on self-reported data for outcomes such as use of contraception and number of pregnancies which may be subject to reporting bias. We also lacked data on women's menstruation status therefore we cannot exclude women who are postpartum amenorrhoeic or menopausal at later cohort points from our unmet need calculations. However, our first collection point for reproductive health questions was 12 months post-partum and lactation amenorrhoea as a contraceptive method should not be considered effective more than 6 months post-delivery (Kennedy and Visness, 1992; Van der Wijden and Manion, 2015). Evidence from other rural regions of the country showed an important reason for women not using contraception in the late postpartum period is the erroneous belief that they are not a risk of pregnancy if their periods have not returned (Tegegn *et al*., 2017; Embafrash and Mekonnen, 2019). In order to avoid capturing women that may become menopausal during the study we restricted upper age of cohort to 38 years. Mean age for menopause is 51 globally however there are large geographical differences (Melby *et al*., 2005). There is limited data on average age of menopause in Ethiopia but regional studies have reported it to be around 42.5 years (Yisma *et al*., 2017) and 45.3 years (Kibret and Mariam, 2019), therefore lower than in other nations.

For the contraceptive outcomes, the CMD exposure does not capture the longitudinal course of mental ill-health. In the analysis of CMD in relation to subsequent pregnancies, exposure to CMD over time is captured through measurement at repeated time points, but we do not have data to investigate the effect of CMD in the perinatal period *v*. at other times on subsequent pregnancies. Also, we cannot exclude that other confounders not used in our models which may play a significant role in our hypotheses. For example, we lacked data on breastfeeding which may be an important mediator in some of the associations studied. We also did not use a standardised and validated measure of social support. However, previous qualitative study with this cohort indicated that the most important source of support for women is from their partner (Hanlon *et al*., 2009a, b) and we therefore adjusted for marital discord. We were unable to explore reasons behind contraceptive failure in our sample. We also lacked information about what other medications, including psychotropic medication, the women may be receiving. However, it is expected that psychotropic medication would be very rarely accessed by study participants given the non-availability of appropriate services at the time when the study was conducted.

### Implications

With the ongoing global efforts to mitigate unmet need for family planning we believe our results provide important evidence to support integration of mental health and physical health services as well as identification of vulnerable populations that are hard to reach within the current models of care. Studies from high-income countries and mostly from clinic-based samples have previously highlighted an association between depression and/or stress and contraceptive non-use and use of less effective methods (Garbers *et al*., 2010; Hall *et al*., 2013a, b). In those settings, suggested mechanisms underlying this association are that psychological distress may negatively impact the decision making and risk assessment abilities of women with regards to contraceptive behaviour (Yuen and Lee, 2003; Hall, 2012). An alternative possible mechanism is that women with CMD symptoms may be more susceptible to perceive somatic side effects of hormonal contraception and therefore discontinue use more readily (Hall *et al*., 2012). Conversely, recent evidence from a high-income country also suggests women who develop postpartum psychiatric disorders are less likely to go on to have further children (Liu *et al*., 2020). We did not find any studies examining the effect of symptoms of depression and anxiety on family planning or fertility in low- or middle-income country settings. Contextual differences may mean that other mechanisms underlie the association between mental health and family planning. In the rural, low-literacy setting of this study, there are many structural barriers to accessing family planning, in particular due to low SES and low empowerment of women. Poorer mental health in a woman may lead to social withdrawal, decreased exposure to health education messages and diminished motivation or capacity to mobilise support to access family planning services. Although an association was found between CMD symptoms at 12 months and unmet need for contraception at 2.5 years, no significant association was found for CMD symptoms at 3 years and unmet need at 3.5 years or between CMD symptoms and number of pregnancies during the study period. Therefore, the relationship between CMD symptoms and adoption of family planning may have a temporal element and be affected by the stage of a woman's reproductive life. Further studies are required to explore these potential mechanisms and inform intervention development.

Our results highlight a need to take a more holistic and person-centred approach to family planning services, including the identification and support of women experiencing psychosocial distress. The World Health Organization mental health Gap Action Programme (mhGAP) seeks to integrate mental health care into all general health care services (WHO, 2008). Although this should include family planning services, there has been limited consideration of the importance of mental health in this area to date. The Ministry of Health of Ethiopia has been supporting integration of services into primary care by issuing a National Mental Health Strategy (Ethiopia FMOH, 2012b) based around mhGAP (WHO, 2013). In addition, mental health is now one of the packages of care for level IV HEWs (Tilahun *et al*., 2017). However, the extent to which HEWs are implementing mental health care is limited by a lack of integration of mental health into supervision structures or the health management information system (Hanlon *et al*., 2017). Effective brief psychological treatments have been developed for CMD symptoms in LMICs and most of these are delivered by community health workers and have been developed for women (Singla *et al*., 2017). The HEP in Ethiopia with its strong community focus and delivery of home visits by female HEWs would, therefore, be well equipped to identify women who might be experiencing mental health symptoms and would benefit from these interventions but may not access other parts of the health system.

## Conclusion

To our knowledge this was the first study to investigate the impact of poor mental health on unmet need for contraception and fertility rate in a rural sample of women from a lower income country. We showed that high level of CMD symptoms at 1 year postpartum is associated prospectively with higher unmet need for contraception independently of SES and educational status. There is a lack of models of care promoting integration of mental and physical health in the family planning setting and further research is necessary to study the burden of preconception mental disorders and how these can be best addressed.

## Availability of data and materials

The dataset included for the analyses within this manuscript can be obtained on request from the corresponding author.
